# A study on digital tooth preparation assessment software in undergraduate pre-clinical skills teaching

**DOI:** 10.1038/s41405-024-00279-4

**Published:** 2024-12-05

**Authors:** Hanin Alsharif, Richard Boyle, Pauline Maillou, George P. Cherukara

**Affiliations:** 1 Resident Dentist – Prosthodontics Tabuk Health Cluster Specialist Dental Center, Tabuk, Saudi Arabia; 2 Lecturer in Digital Dental Technology, University of Dundee, Dundee, UK; 3 Clinical Senior Lecturer and Honorary Consultant in Restorative Dentistry, University of Dundee, Dundee, UK; 4 Senior Clinical Lecturer, University of Dundee, Honorary Consultant in Restorative Dentistry, NHS Tayside, Programme Director, DClinDent Prosthodontics, School of Dentistry, University of Dundee, Park Place, Dundee, DD1 4HN Scotland UK

**Keywords:** Dentistry, Restorative dentistry

## Abstract

**Introduction:**

Aims This research aims to evaluate the effectiveness and efficiency of the PrepCheck digital system as an additional feedback tool in enhancing undergraduate dental students’ tooth preparation skills and its potential to enhance students’ learning experience.

**Material and Methods:**

A total of 55 BDS3 students attending the “Crowns Course” and divided into three groups participated in the study. One group (*n* = 24) was randomly selected as the case group and received feedback using the digital tooth preparation analysis system, PrepCheck, alongside standard visual assessment. The other two groups (*n* = 31) served as controls and only received standard visual feedback. All students’ tooth preparations for the final test were digitally assessed using PrepCheck against a faculty-approved master preparation. The tooth preparation quality was compared between the case and control groups, employing two distinct grading methods. Additionally, a questionnaire was provided to students who used the digital system to gather their feedback.

**Results:**

The findings revealed a positive trend in performance among the case group when using the PrepCheck system. However, the analysis showed no statistically significant differences between the groups in both the tutor assessment only and tutor assessment in addition to using the PrepCheck report. Despite the absence of statistically significant differences, qualitative feedback from participants indicated a favourable reception of the digital system.

**Conclusions:**

While the PrepCheck digital system displayed potential in complementing traditional teaching methods and enhancing the learning experience, its integration posed challenges, particularly concerning time constraints. Further research is recommended to investigate further the potential longer-term effects and potential useful applications for integrating digital systems like PrepCheck into dental education.

## Introduction

Dental education relies heavily on developing hands-on skills, especially in fields like fixed prosthodontics, where practical competencies are essential for restoring dental function and aesthetics [1]. Fixed prosthodontics involves understanding biomechanical principles, biomaterials, and having practical clinical techniques to restore teeth functionally and aesthetically [1, 2]. Before providing clinical care for patients, students are expected to successfully demonstrate competence in relevant clinical skills in simulated laboratory settings. These facilities and management of them often has resource and time constraints [3].

**Fig. 1:**
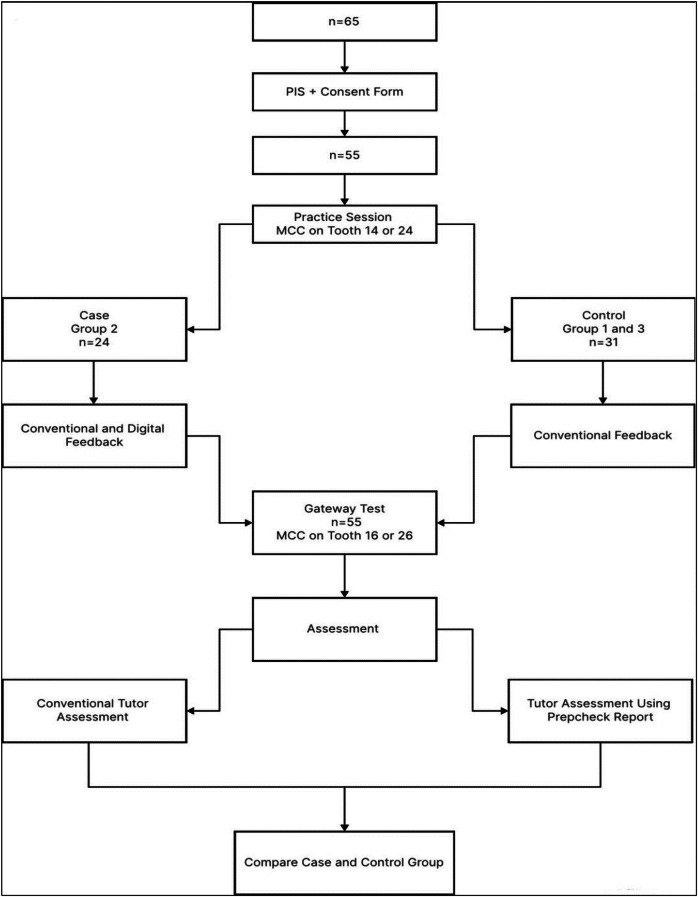
Summary of the study sequence.

There are two conventional and popular ways in which students receive feedback on their work. Tutor-directed clinical skills laboratory sessions are the standard approach for teaching clinical dental skills [4]. In addition, the students may visually receive feedback by comparing their efforts to the starting shape using an index/template and applying their theoretical knowledge. Students (directed by their tutor may create a pre operative index/template using, for example silicone putty of unprepared morphology (see Fig. 1) [5]. Assessment of a student’s work by the tutor may have an element of assessor subjectivity that can lead to inconsistency and hence confusion. However, visualising (as described in the previous paragraph) and feedback by the students using templates have been reported to be inaccurate in assessing tooth preparation quality [6].

Feedback provided to students in preclinical dental courses has been found to be slow, inconsistent, and subjective [7]. Survey findings reveal that students perceive inconsistent feedback as having a negative influence on their learning journey [8–10]. These facts, even in well-supported teaching, slow down and diminish students’ learning experience and acquired proficiency [11]. Therefore there may be a need for more objective and reliable assessment methodologies [12]. Nevertheless, Stoilov et al. [11] reported a strong student preference for feedback from tutors over digital assessment tools.

Digital advancements have progressively reshaped dental education, transitioning from early simulation tools reliant on mouse or keyboard input to more sophisticated and interactive technologies. The earlier methods fell short in helping learners hone basic sensory motor skills. However, the advent of virtual reality, haptic (tactile), and robotic technologies has led to significant progress in dental education [13]. Supplementing conventional verbal and visually comparative feedback with digital, visual and colour coded information is anticipated to improve students’ understanding of the feedback components, improve consistency and hence help them to effectively act on the feedback to allow improvement to at least the acceptable standard [12].

Digital dental education utilising digital evaluation systems such as PrepCheck (Dentsply Sirona, Germany), Dental Teacher (KaVo, Germany), and Compare (Planmeca, Finland) are commonly used in dental training settings, mainly to assess students’ skills [7, 11, 14]. It is reported that digital systems and software may help improve education and assessment, especially for dental students during their early training. It has been reported that the utilisation of digital tools in learning has rendered the process of acquiring knowledge and monitoring progress more efficient, reliable, and accessible [11].

One such tool, the PrepCheck system (Dentsply Sirona, Germany), enables digital analysis of students’ tooth preparations against a standard master model. This system uses geometric analysis, providing visual feedback that highlights areas needing improvement and facilitating a more reliable self-assessment process. Studies have suggested that integrating digital systems like PrepCheck could complement traditional teaching, enhancing objectivity in assessments and supporting students’ skill development in preclinical settings. This study examines the effectiveness of PrepCheck as an additional feedback tool to enhance the quality of student tooth preparation and improve the overall learning experience [12]. However, it is noted that digital assessment tools can only partly replace traditional assessment methods, indicating that a blend of digital and conventional assessments might be the most effective way to train and evaluate dental students [15].

The literature consistently underscores escalating trust in digital tools, such as PrepCheck and intraoral scanners, for reshaping dental education. While offering objective feedback and enhancing self-assessment capabilities, these tools do not reduce the value of tutor feedback. The convergence of traditional and digital pedagogical strategies appears to offer the most holistic approach to dental education.

This study aims to investigate the effectiveness of PrepCheck, as additional feedback to conventional feedback methods to provide undergraduate students with enhanced feedback on tooth preparation assessment and a better student learning experience. In addition, the study aimed to measure how students feel about using this technology alongside traditional instruction and feedback. The null hypothesis for this study is that there is no difference between digital and/or conventional tooth preparation evaluation and feedback methods during teaching, the resulting quality of the students’ preparation in the practical assessment test, or their learning experience.

This study is important as it informs on the potential of improving the quality of teaching and learning using PrepCheck, which could influence students’ competency acquisition, thereby potentially influencing patient safety and care quality. This study addresses the question: ‘Does the digital tooth preparation assessment system and resulting feedback enhance the quality of students’ tooth preparation and give undergraduate students an improved learning experience?’

The hypothesis tested in this study is that the use of digital feedback through PrepCheck will lead to a significant improvement in students’ preparation quality and learning outcomes compared to traditional assessment methods alone.

## Methods

### Ethical consideration

Informed and written consent was obtained from all study participants (case and control groups). This sequential provision of information and collection of consent ensured ethical and transparent engagement with the participants, in alignment with the ethical approval and guidelines stipulated by the Ethics Committee of the University of Dundee (EA No. UOD-SREC-SDEN-2023-005).

### Research approach

This study employed a mixed-methods research approach to provide a comprehensive analysis of the impact of digital feedback on the quality of students’ tooth preparation. The participants consisted of third-year dental students undertaking clinical skills teaching of tooth preparation for metal-ceramic crowns, following completion of a theoretical related course on crown preparation, who provided informed consent to participate in the study, and had no previous experience of tooth preparation for indirect restorations were included in this study.

### Sampling and grouping

**Inclusion Criteria:**

- Third-year students enrolled in the “Crowns Course” during the 2022–2023 academic year.
- Successfully completed a preliminary theoretical course on crown preparation.
- Willing to provide informed consent to participate in the study.
- No prior experience with tooth preparation.

**Study Groups:**

1. **Case Group (*n*** = 24):

- Received both conventional feedback and additional digital feedback using the PrepCheck system.

2. **Control Groups (*n*** = **31):**

- Group 1 (*n* = 20): Received only standard verbal-visual feedback using the SMART Visualizer.
- Group 3 (*n* = 11): Also received only standard verbal-visual feedback using the SMART Visualizer.

### Study sequence

The study sequence is summarised if Fig. 1.

### Preparation comparison

**Fig. 2:**
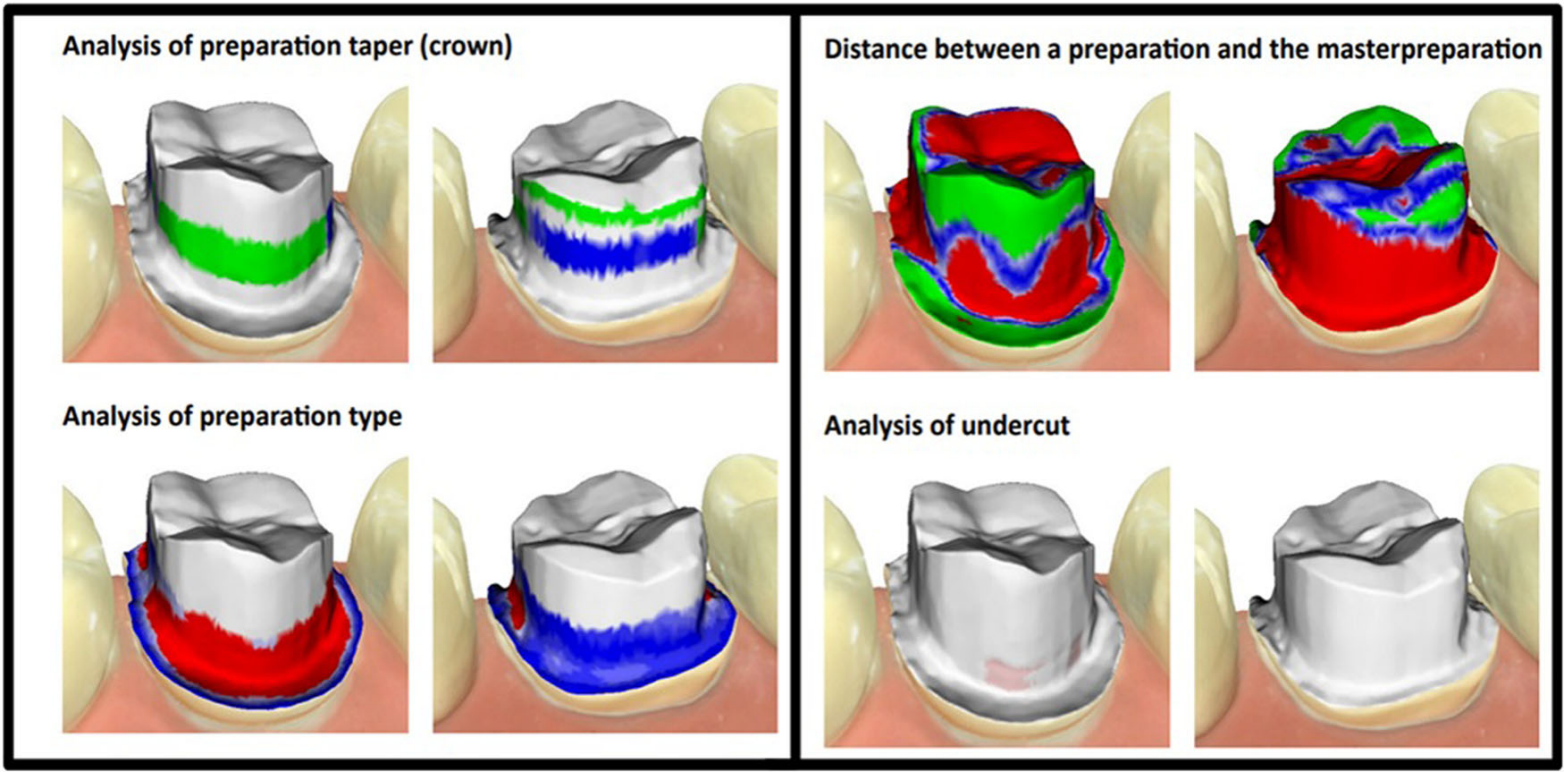
The PrepCheck software contrasts a student’s tooth preparation with a ‘Master Preparation’ using a colour-coded system: Green: Indicates areas needing improvement but can still be rectified. Blue: Denotes the optimal alignment with Master Preparation. Red: Highlights areas that deviate and cannot be improved.

The tutor prepared a faculty-approved master’s preparation for scanning into PrepCheck, especially in the present research. All student preparations were then compared to it. The superimposition of student preparations with faculty-approved master preparation comparisons provides a detailed analysis. Aspects of the preparations that were compared by PrepCheck included the depth of axial and occlusal reduction, the taper of the preparation in buccolingual and mesiodistal aspects, the presence of undercuts, type, and width of the finish line, and the nature of the line angles of the preparation. Figure 2 illustrates an example of a report provided by the tutor for the students after analysing their preparation using the PrepCheck system.

### Evaluating the impact of digital feedback on tooth preparation

Comparisons of the quality of the tooth preparations submitted by students who were taught with feedback using the digital system (cases, *n* = 24) and those who did not use the digital system (controls, *n* = 31) were made. The final grade for preparing metal-ceramic crowns for each student was used as a measure of their acquired skill level over the course of their classes.

### Assessment procedures

#### Conventional

The quality of the preparations was assessed using a scoring system devised by the experienced tutors. The evaluation criteria were based on clinical parameters for successful preparation. The test preparations were assessed by three experienced tutors, each of whom taught a separate class (groups 1 to 3). However, students were graded blindly with one tutor assessing only one student’s work. There was no second grade; therefore, this was the student’s final grade. The grades were based on established clinical and academic criteria.

Digital scans were created for all student preparations in both the case and control groups at the final assessment. These preparations were evaluated blindly by the case group tutor through a digital report generated by PrepCheck software. The tutor produced a grade for each scan using the same criteria as the preparations were graded by. This software facilitated digital analysis of the students’ molar preparations and compared them with tutor’s master preparations. This digital evaluation report was completed after the examiners marked the test and had no impact on the students’ final grades.

### Student’s views

Questionnaires were used to understand what the students thought of using PrepCheck. To make the students’ subjective assessments amenable to statistical analysis, answers were recorded on a scale (strongly agree, agree, neutral, disagree, strongly disagree) that could easily be quantified. Qualitative data were collected through questionnaires to gather the participants’ perspectives and feedback regarding the teaching methods employed, particularly the use of the digital tooth preparation analysis system.

### Data collection methods

Data were collected in multiple ways.

1. Grade from the standard visual assessment of the tooth preparations submitted during the final test.
2. Grades from the direct scanning of tooth preparations and digital analyses were performed using PrepCheck software.
3. Questionnaire feedback from the case group regarding the digital analysis system.

### Outcome measures

The primary outcome measure was the quality of the tooth preparations assessed against tutor-approved optimal preparation dimensions using the report generated by the PrepCheck software. The assessment of the tooth preparation quality was categorised as “acceptable”, which represents the ‘good’ and ‘competent’ quality grades or “not acceptable”, which represents learner quality grade based on the preparation’s adherence to teaching standards. The secondary outcome was the perceived utility and effectiveness of the digital analysis system, gauged through the questionnaire.

### Statistical analysis

Descriptive statistics is presented in form of mean, standard deviation. Frequency and percentages. For statistical analysis a software statistical package for social sciences (SPSS version 29, IBM, Armonk, NY, USA) was used. Maan Whitney U test was applied to check the difference between case and control participants’ responses. The significance level was set at *p* < 0.05.

## Results

In this study, a total of 55 participants were included. The participants were divided into three groups: two control groups, comprising 31 students (56.36%), and the case group, comprising 24 students (43.64%), as shown in (Table 1).

**Table 1:** Tutor grade evaluation.

	Group	N	Mean Rank	Sum of Ranks	Mann-Whitney U	*p*-value
Tutor	Control	31	27.55	854.00	358.000	0.697
	Case	24	28.58	686.00		
	Total	55				

The data in Table 1 reveal no significant deviation between the control and case groups (U = 358.000, *p* = 0.697); the *p*-value of 0.697 signifies that it is not statistically significant.

As illustrated in Table 2, there is a trend towards higher rank distributions in the case group compared to the control group (U = 319.500, *p* = 0.274), the *p*-value of 0.274 reveals that it is not statistically significant.

**Table 2:** PrepCheck grade evaluation.

	Group	N	Mean Rank	Sum of Ranks	Mann-Whitney U	*p*-value
PrepCheck	Control	31	26.31	815.50	319.500	0.274
	Case	24	30.19	724.50		
	Total	55				

Table 3 provides the comparison between tutor and PrepCheck assessments did not show statistically significant differences between the case and control groups. Tutor evaluations resulted in a mean score of 2.13 ( ± 0.341) for the control group and 2.17 ( ± 0.381) for the case group, while PrepCheck assessments yielded mean scores of 1.87 ( ± 0.499) for the control group and 2.04 ( ± 0.624) for the case group.

**Table 3:** Descriptive statistics of Tutor and PrepCheck assessments.

	Control (*n* = 31) Mean ± SD	Case (*n* = 24) Mean ± SD
Tutor	(2.13 ± 0.341)	(2.17 ± 0.381)
PrepCheck	(1.87 ± 0.499)	(2.04 ± 0.624)

Comparison of mean grades between the case and control groups, assessed using two distinct methods: a conventional tutor assessment and a tutor assessment utilising the PrepCheck system. The Case group, which received additional digital feedback through the PrepCheck system, exhibited enhanced performance in their tooth preparation test compared to the control group across both assessment methodologies. Specifically, the Case group secured a mean grade of 2.17 in the Tutor assessment and 2.04 in a Tutor assessment employing PrepCheck, while the Control group attained mean grades of 2.13 and 2.04, respectively in the same assessments.

**Fig. 3:**
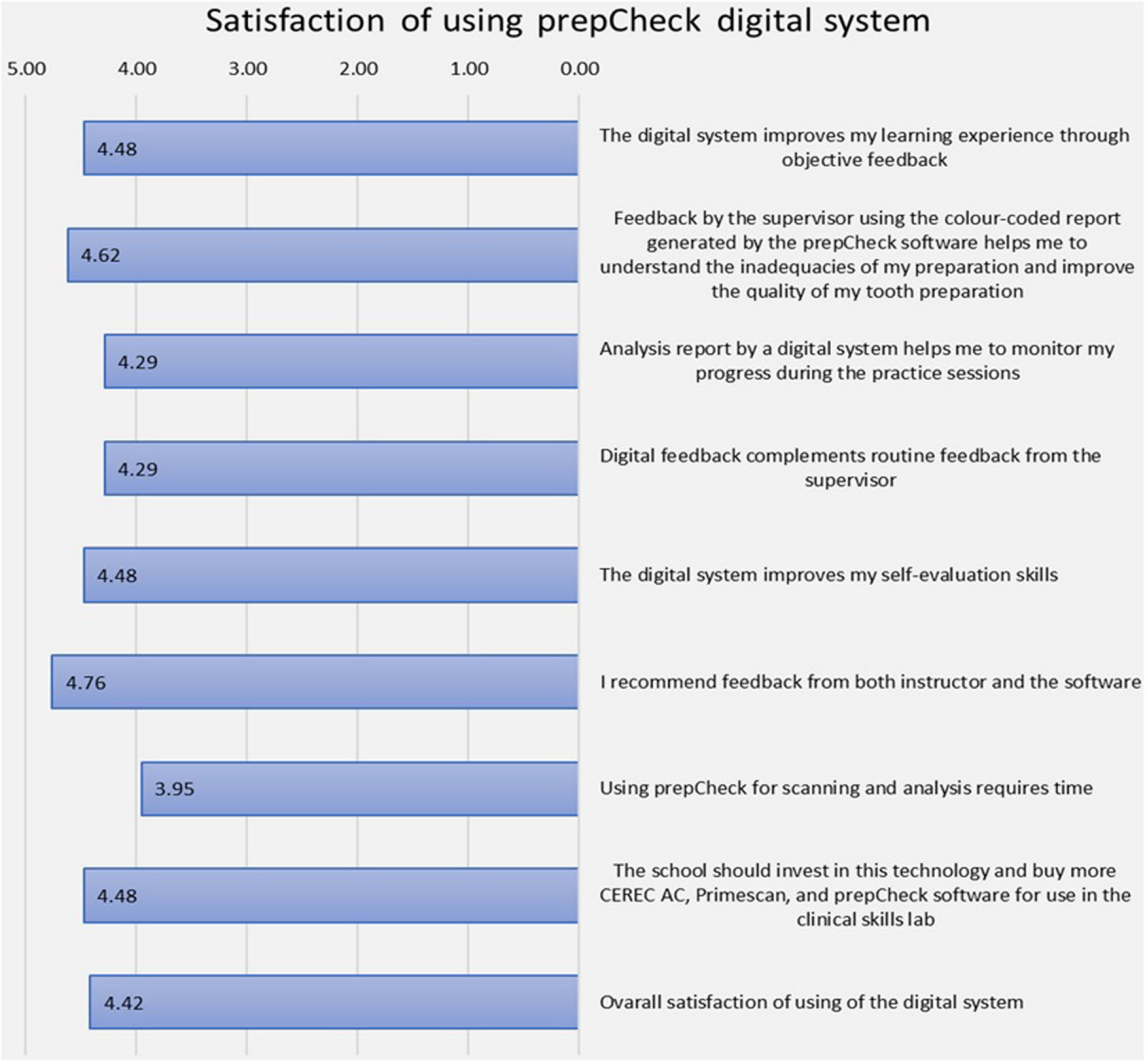
Satisfaction of using PrepCheck digital system.

In the bar chart (Fig. 3), the provided data reflect the participants’ satisfaction levels concerning PrepCheck digital system usage. With a mean overall satisfaction score of 4.42 out of a possible 5, there was a consensus of high satisfaction among the participants regarding the use of the PrepCheck digital system to enhance their tooth preparation skills. This high level of satisfaction, with a mean score close to the maximum of five, underscores the positive reception and effectiveness of the PrepCheck digital system as perceived by the participants.

## Discussion

This study aimed to investigate whether a digital feedback tool, PrepCheck, could supplement conventional instruction and feedback to improve the skill acquisition of students and how students would feel about using it. PrepCheck appeared to improve the quality of tooth preparation among the case group, showing a positive trend in their performance with the use of this digital tool. Furthermore, the study’s questionnaire results showed a favourable reception of the digital system. There was an evident appreciation among the participants regarding the potential benefits of digital interventions in their learning process. However, when we look at the statistical analysis, the difference in grades between the case and control groups was not statistically significant enough to prove that the PrepCheck system had a major impact on improving students’ tooth preparation quality.

This scenario presents a complex picture. On the one hand, students demonstrated a visible improvement in performance with PrepCheck, but on the other hand, the statistical data did not validate this improvement. This situation highlights the need for a deeper understanding of how digital tools, such as PrepCheck, interact with teaching methods and what other factors might influence the results. Although technology may have the potential to enhance learning and teaching, its impact is inevitably influenced by other factors. As we discuss the broader implications of this study, it is important to consider a wider range of factors that might affect how digital tools can be effectively integrated into dental education to improve the learning experience and quality of dental practice.

Are there pedagogical approaches that yield stronger, statistically significant results? Even if so, practical circumstances still hold here, however. Can the financial and time investment needed to make a pedagogical approach return increased skill acquisition be justified within the constraints of delivering a course in real life and the trade-offs an actual department would need to make?

Several studies have explored the advantages and limitations of digital assessment tools in tooth preparation. Hamil et al. [7] were early proponents of digital tools in dental education, emphasising the merits of the E4D Compare software for student self-assessment. Their work established a precedent, suggesting that such digital tools could empower students to quickly identify deficiencies in their work, furthering the argument for integrating these tools into dental curricula.

The literature shows that the integration of digital feedback systems into dental pedagogy has been a point of contention and interest in recent years. Jorquera et al. [16] ventured into the realm of CAD/CAM technology and its application in student training. Their study accentuated the benefits of digital technology in both preclinical and clinical environments, aiding students in recognising preparation flaws. E4D Compare software has emerged as a potent tool, according to the endorsement of some researchers. Hamil et al. [7] emphasised its efficacy in enhancing intra- and inter-grader agreement during grading in a student simulation laboratory. Similarly, Park et al. [17] highlighted the potential of the PrepCheck software in the preclinical prosthodontics domain. Their findings revealed that the students perceived this tool as an invaluable asset for self-assessment. Further, Seet et al. [15] investigated the potential of the 3 Shape TRIOS intraoral scanner in student crown preparation evaluation. Their research demonstrated the reliability of digital tools in offering granular and precise assessments. Miyazono et al. [18] corroborated this sentiment by highlighting the improved intra- and inter-grader agreement when deploying digital scanning and tooth preparation assessment software compared to traditional grading paradigms.

Our study found no significant difference in outcomes in the quality of the students’ tooth preparation based on condition (case vs. control). However, the overarching theme in the literature underscores the pivotal role of the assessment method itself. While traditional methods offer their unique benefits, digital tools, as consistently evidenced, present a more objective, detailed, and reliable evaluation mechanism.

The lack of statistically significant findings in our study echoes some of the sentiments found in the literature. For instance, Nothaft et al. [19] emphasised that while digital tools such as PrepCheck were viewed positively, they could not replace tutor feedback (although this study sought to complement tutor feedback). This point is further bolstered by the findings of Stoilov et al. [11], who found that students preferred direct feedback from faculty staff over digital validation systems.

Some researchers argue for the utility of digital tools in supporting, rather than replacing, traditional methods. Findings from the study by Schepke et al. [12] emphasised that teaching dental skills can be notably improved by incorporating PrepCheck into the assessment procedure. In their study, instructors were considerably more in agreement when using PrepCheck for assessment. This aligns with our understanding of the potential of digital tools to enhance objectivity in evaluations and to complement tutor feedback. The results of Schepke et al. [12] further suggested that using PrepCheck effectively aids students in learning practical dental skills. They emphasised the importance of students learning to work with modern digital technologies, especially given the trajectory of these technologies becoming increasingly prevalent in dental practices. Interestingly, Schepke et al. [12] also found that, despite the application of the same evaluation criteria, there was a marked difference in instructor assessments when comparing conventional methods with PrepCheck, underscoring the tool’s ability to enhance inter-assessor agreement. Furthermore, feedback provided by PrepCheck was perceived as consistent, objective, and accurate by the students, bolstering their preparation practices. However, they also highlighted a crucial challenge, much like our study: the time-intensive nature of the scanning process. This, in turn, limits the students’ preparation time for examinations. Despite these challenges, the students viewed PrepCheck as a desirable feedback tool, indicating its potential to reshape practical dental skill pedagogy.

Considering these insights and the added perspectives of Schepke et al. [12], it is evident that while digital tools offer potential improvements in dental education, their integration presents challenges that need to be addressed to harness their full potential. The research conducted by Kunkel et al. [20] argues that digital grading through PrepCheck furnished a more objective and precise assessment compared to the subjective grading proffered by instructors, which was inclined towards inflation. The precision and objectivity offered by PrepCheck accorded students immediate and accurate feedback regarding the quality of their preparations, although it did not directly enhance tooth preparation quality. Instead, emphasis was placed on the accuracy and promptness of rendered feedback. It could be that what is needed is not a more accurate tool but a validated theory of change for improving skill acquisition. Perhaps a digital feedback tool could be effectively deployed in this context to see verifiable increases in skill acquisition.

This study faced several limitations that may have influenced the results. First, the relatively small sample size (*n* = 55) limited the statistical power, potentially explaining the lack of significant differences between the case and control groups. Additionally, the short exposure to the PrepCheck system and limited practice time may have restricted students’ ability to fully benefit from digital feedback. Students were only given a brief period to familiarize themselves with the tool, which may have affected their skill development. The study was also subject to time constraints in the laboratory, which may have limited the opportunities for students to engage with both traditional and digital feedback methods adequately. Finally, variability in feedback style among tutors, despite standard grading criteria, could have introduced slight inconsistencies in assessment, impacting the uniformity of results. Addressing these limitations in future studies—by using larger sample sizes, longer exposure times, and standardized feedback delivery—may provide a clearer understanding of the digital tool’s impact on student outcomes.

## Conclusion

This study suggests that while the PrepCheck digital system shows promise as a supplementary tool for enhancing dental students’ tooth preparation skills, the results did not demonstrate statistically significant improvements over traditional feedback alone. Positive student feedback indicates potential benefits in learning engagement, though challenges with time and system integration remain. Future research with larger sample sizes and extended exposure to digital tools is recommended to explore their long-term impact on skill development in dental education.

## Data Availability

The original data is presented in the study are included in the article, further inquiries can be directed to the corresponding author.
